# Elevational Distribution and Ecology of Small Mammals on Tanzania's Second Highest Mountain

**DOI:** 10.1371/journal.pone.0162009

**Published:** 2016-09-21

**Authors:** William T. Stanley, Philip M. Kihaule

**Affiliations:** 1 The Field Museum of Natural History, Department of Science and Education, Chicago, Illinois, United States of America; 2 University of Dar es Salaam, Department of Zoology, Dar es Salaam, Tanzania; University of Sydney, AUSTRALIA

## Abstract

Mt. Meru is Tanzania’s second highest mountain and the ninth highest in Africa. The distribution and abundance of small mammals on this massif are poorly known. Here we document the distribution of shrews and rodents along an elevational gradient on the southeastern versant of Mt. Meru. Five sites were sampled with elevational center points of 1950, 2300, 2650, 3000, and 3600 m, using a systematic methodology of standard traps and pitfall lines, to inventory the shrews and rodents of the slope. Ten species of mammal were recorded, comprising 2 shrew and 8 rodent species with the greatest diversity for each group at 2300 m. No species previously unrecorded on Mt. Meru was observed. Two rodent genera that occur in nearby Eastern Arc Mountains (*Hylomyscus* and *Beamys*) were not recorded. The rodent *Lophuromys verhageni* and a recently described species of shrew, *Crocidura newmarki*, are the only endemic mammals on Mt. Meru, and were widespread across the elevational gradient. As in similar small mammal surveys on other mountains of Tanzania, rainfall positively influenced trap success rates for shrews, but not for rodents. This study provides new information on the local small mammal fauna of the massif, but numerous other questions remain to be explored. Comparisons are made to similar surveys of other mountains in Tanzania.

## Introduction

The distribution of mammals along mountain slopes is of increasing interest to ecologists and mammalogists to document species turnover along environmental gradients and, as a result, the efforts to document the montane faunas of various massifs around the world have intensified over the past few decades. Climate change has recently increased this curiosity and the need for detailed investigations concerning this subject. Documenting the present elevational distribution of organisms along a given slope will facilitate the monitoring of that biota during times of climatic perturbation or habitat alteration. Biogeographic, ecological, and evolutionary studies are also advanced by a greater comprehension of montane biotic systems. Examples of elevational surveys of small mammals in montane localitiesusing systematic sampling protocols include Chile [1], Costa Rica [2], Madagascar [3], Malaysia [4], Philippines [5], Taiwan [6], and U.S.A. [7]. Each of these studies produces a more complete understanding of both specific and broad patterns of mammalian elevational distribution, and the mechanisms that led to such an array. [8]. The utility of such studies in monitoring impacts of environmental change through time cannot be overstated. For example, in Yosemite Valley, California, a survey along an elevational transect documented significant range shifts in various mammalian species since an identical survey was conducted along the same transect a century earlier [7].

The montane mammals of sub-Saharan Africa have been studied for over a century, and research concerning their elevational distribution on some massifs is well documented [9], [10]. Published results from detailed systematic inventories of mammals on mountains of eastern Africa include, Kerbis Peterhans et al. [11] for the Rwenzori Mountains, Stanley and Hutterer [12] for the Udzungwa Mountains, and Mulungu et al. [13] and Stanley et al. [14] for Mt. Kilimanjaro. Certain other topographically important massifs remain enigmatic with regards to the mammals occurring on them.

Mt. Meru, Tanzania’s second highest mountain, and the ninth highest in Africa, is a case in point. While its neighbor, Mt. Kilimanjaro, has been the subject of several studies on the locally occurring mammal fauna [13], [14], [15], [16], Mt. Meru remains understudied. While some aspects of the ecology of the mountain have been documented [17], the mammal fauna has been largely neglected. The most complete analysis of the fauna of Mt. Meru to date is that of Demeter and Hutterer [18], but is not based on any standard trapping protocol and several different habitats on the massif are not included. The need for more detailed information of this and nearby mountains has been emphasized by the results of other studies [19], which indicate that climate change is affecting the ecology and habitat of neighboring Mt. Kilimanjaro. Baseline data for the small mammals of Mt. Meru will provide the means for future analyses of the impact of climate change on the ecology of this volcano.

We used a standardized methodology that has been recently employed in myriad other montane sites of Tanzania [12], [20], [21], [22] to survey the small mammals (shrews and rodents) at five different elevations and habitats along the southeastern slope of Mt. Meru. Our study has three principal goals: 1) to conduct the first intensive survey of the presence and abundance of small mammals along an elevational gradient on the mountain; 2) to test for differences between rodents and shrews in their elevational distribution, their response to different trapping methodologies, and the relationship of captures to rainfall patterns; and 3) to compare the generated results to similar studies on other mountains Tanzania.

## Materials and Methods

### Study site

**Fig 1:**
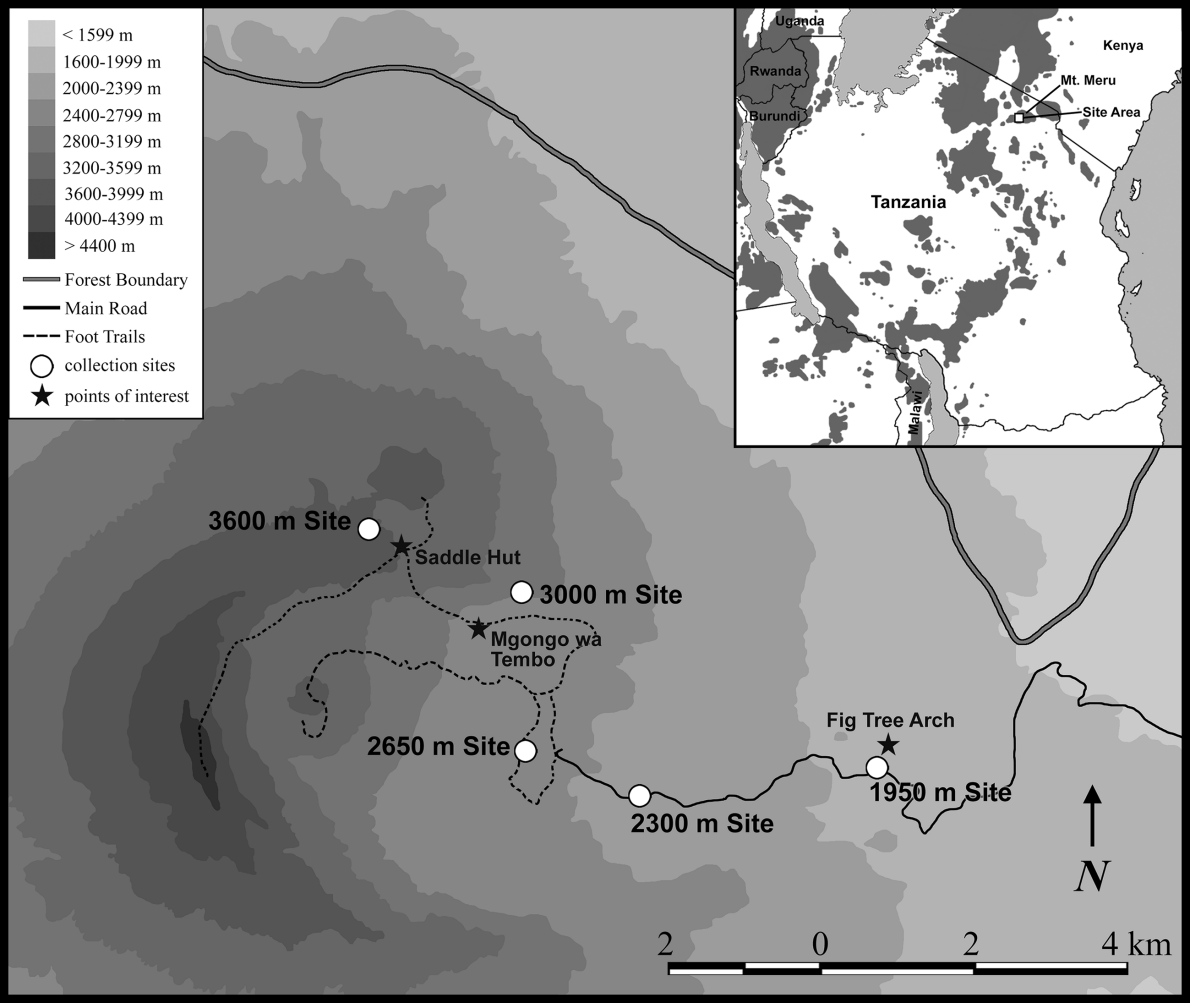
Map of Mt. Meru showing routes, elevational contours, and study sites.

Mt. Meru is in northeastern Tanzania and reaches an elevation of 4,566 m (14,977 ft), and ranks ninth among the 10 highest mountains of Africa. An active volcano (the mountain last erupted in 1910), Mt. Meru is the centerpiece of Arusha National Park The mountain is a popular destination for climbers, and there is one path that originates in the lowlands and runs up the southeastern side of the mountain [18]. Between 16 July and 19 August 2009, we sampled the small mammals (shrews and rodents) at five different elevations, ranging from roughly 1950 to 3600 m, along the climbing route on the southeastern slope of Mt. Meru (Fig 1).

All sampling sites were on Mt. Meru in Arusha National Park, Arumeru District, Arusha Region, Tanzania. The specific localities, elevations, habitats (sensu Demeter and Hutterer [18]), and dates of sampling are listed below. Using a minimum-maximum thermometer and a mobile rainfall gauge, measurements of temperature and precipitation were collected on a daily basis at each respective camp; these data are summarized in Table 1. The elevations given for each site are centered at the associated camp and sampling efforts spanned roughly 100–200 m above and below the camp:

Site 1–1950 m)—Fig Tree Arch, 3.24406°S, 36.82845°E, 1950 m; lower montane forest; 16–23 July 2009.

Site 2–2300 m)—Site 2, 3.24725°S, 36.80066°E, 2300 m; upper montane forest; 23–30 July 2009.

Site 3–2650 m)—Meru Crater, 3.242°S, 36.78736°E, 2650 m; ecotone between montane forest and ericaceous zone; 13–19 August 2009.

Site 4–3000 m)—Mgongo wa Tembo, 3.2235°S, 36.78675°E, 3000 m; mix of forest, ericaceous plants, and some bamboo; 30 July-6 August 2009.

Site 5–3600 m)—near Saddle Hut, 3.21609°S, 36.76897°E, 3600 m; ecotone between ericaceous and alpine zones; 6–13 August 2009.

**Table 1:** Climatic data registered at each camp site on Mt. Meru in July-August 2009 in the context of an elevational survey of small mammals. Totals presented as mean ± standard deviation, range, and sample size (number of days measured). Rainfall samples are given as number of days monitored and (number of days with rain).

Elevation (m)	Daily minimum temperature (°C)	Daily maximum temperature (°C)	Daily rainfall (mm)
1950	10.2 ± 0.4	16.7 ± 1.0	0.7 ± 1.2
	10–11	16–18	0–3
	N = 7	N = 6	N = 7 (2)
2300	7.9 ± 0.9	14.2 ± 1.8	0
	6.5–9.0	11.5–16	
	N = 7	N = 6	N = 6
2650	6.2 ± 0.7	13.1 ± 2.7	0
	5.5–7.5	10–18	
	N = 6	N = 6	N = 6
3000	4.3 ± 0.9	13.8° ± 3.4	3.0 ± 3.3
	3–5	9.0–16.5	0–6.2
	N = 7	N = 6	N = 6 (3)
3600	2.1 ± 1.4	16.3 ± 1.6	0
	0–4	14.5–18.0	
	N = 7	N = 6	N = 6

### Field methodology

Pitfall lines and traplines were installed to capture principally shrews and rodents, respectively. Each pitfall line were comprised of 11 buckets, placed 5 m apart, and buried in the ground so that the top of the bucket was level with the ground. Each of these 15 l buckets was 26 cm high and had an upper and lower diameter of 26 cm and 24 cm, respectively. Each pitfall line had a 50 cm high black plastic drift fence running over the center of each bucket. This technique has been used with considerable success in other mammal surveys.

Trap lines utilized three different kinds of traps: Museum Specials, 14 x 7 cm; Victor Rat Traps (referred to here as Victor Trap), 17.5 x 8.5 cm; and medium-sized Sherman Traps, 23 x 9.5 x 8 cm. To maximize capture success, traps were set at sites considered likely to be frequented by small mammals, rather than at fixed distances or in a grid. Consequently, distances between consecutive traps were not constant. Bait for each trap consisted of freshly fried coconut coated in peanut butter, and traps were rebaited each day in the late afternoon. Additional details on these trapping techniques are presented by Stanley et al. [22]. Both pitfall and trap lines were checked twice each day, in the early morning and mid-afternoon, for captured animals.

Not all traps or buckets were employed for equal amounts of time (some trap lines were set the first day of the survey, others were installed on a subsequent day), so we use the terms “trap night”, “bucket night” and “sample night” to quantify sampling effort. A “trap-night” refers to one trap in operation for a 24 hr period (0700 to 0700 hrs). A “bucket-night” denotes one bucket in operation for a 24 hr period (0700 to 0700 hrs). The term “sample-night” is used in reference to overall sampling effort (including the number of trap-nights and bucket-nights). We refer to the success rate of each method as either “trap success” or “bucket success”, and calculate these values by dividing the number of individuals captured by the number of trap-nights or bucket-nights and multiplying by 100. In discussions involving the overall capture success, the term “sample success” refers to the success rate for pitfall and trap lines combined. This is calculated by dividing the number of individuals captured by the number of sampling-nights and multiplying by 100.

Animals were handled following the protocol approved by the American Society of Mammalogists [23]. As all of the species handled are small-bodied, animals were dispatched by cervical dislocation and following AVMA guidelines [23]. Voucher specimens were prepared as either museum study skins with associated skulls and axial skeletons or embalmed in formalin. Standard museum measurements [24] were taken by WTS, and tissues including heart, liver, kidney and/or muscle were extracted from select specimens and frozen in liquid nitrogen, or saved in dimethyl sulfoxide buffer (DMSO) at ambient temperature. All voucher specimens are deposited in the Field Museum of Natural History (FMNH), Chicago, and the University of Dar es Salaam (UDSM), Dar es Salaam, and all tissue samples are in liquid nitrogen storage in the FMNH. We follow the taxonomy presented for shrews by Hutterer [25] and rodents by Carleton and Stanley [26], [27], Holden [28], and Musser and Carleton [29].

### Ethics Statement

Permits for the collection and export of specimens were provided by the Tanzania Commission for Science and Technology (reference no. 2009-134-ER-90-172), the Tanzania Ministry of Natural Resources and Tourism, Wildlife Division (reference no. GD/R.40/1/), and the Tanzania National Parks (reference no. TNP/HQ/C.10/13). Import of specimens into the USA was approved by the US Fish and Wildlife Service (3177-W10414-9/04/09). Shrews and rodents were euthanized following the protocol approved by the American Society of Mammalogists [23], and the study was approved by the Field Museum of Natural History Institutional Animal Care and Use Committee (09–3).

## Results

Over the course of the survey, we accumulated 7,111 sample-nights (4592 trap-nights and 2519 bucket-nights) and captured 751 small mammals, including 276 shrews representing 2 species, and 475 rodents representing 8 species (Tables 2, 3 and 4). Significantly more shrews were captured in buckets than in traps (*X*^2^ = 61.3, P<0.05), and significantly more rodents were caught in traps than in buckets (*X*^2^ = 232.7, P<0.05), a pattern observed in previous Tanzanian small mammal studies employing the same field protocols [12], [14], [30]. In 4592 trap-nights, 581 mammals were captured for an overall trap success of 12.6%. Of the mammals caught in traps, 465 were rodents (10.1% trap success) and 116 were shrews (2.5% trap success). In the accrued 2519 bucket-nights, 170 mammals were captureed for a total bucket success of 6.7%. Of these, 160 were shrews (6.3% success) and 10 were rodents (0.4% success). This conspicuous difference was evident both across the entire survey and at each of the five sites sampled (Table 2). Both shrew species (*Crocidura allex* and *C*. *newmarki*, weighing between 3.5–11 g) found during the survey were caught in traps. Ten rodents representing 3 species (*Dendromus insignis*, *Mus triton*, and *Praomys taitae*) were caught in buckets and ranged in weight 10–27 g. Of the 10 *Dendromus* captured, the lightest (10–13 g; n = 6) were captured in buckets (all adults based on fused cranial sutures), and the heaviest individuals (14–22.5 g; n = 4) were captured in traps.

**Table 2:** Total capture rates for rodents and shrews by trap technique on the southeastern slope of Mt. Meru in July-August 2009.

Elevation	1950 m	2300 m	2650 m	3000 m	3600 m	Totals
**BUCKETS**						
# bucket-nights	506	506	506	506	495	2519
# individuals	52	24	18	63	13	170
(% bucket success)	(10.3)	(4.7)	(3.6)	(12.3)	(2.6)	(6.7)
# species	3	3	3	4	3	5
# shrews	51	22	17	58	12	160
(% bucket success)	(10.1)	(4.3)	(3.4)	(11.3)	(2.4)	(6.3)
# shrew species	2	2	2	2	2	2
# rodents	1	2	1	5	1	10
(% bucket success)	(0.2)	(0.4)	(0.2)	(1.0)	(0.2)	(0.4)
# rodent species	1	1	1	2	1	3
**TRAPS**						
# trap-nights	920	920	920	920	912	4592
# individuals	115	233	104	93	36	581
(% trap success)	(12.5)	(25.5)	(11.3)	(9.3)	(3.9)	(12.6)
# species	5	6	8	9	6	9
# rodents	86	206	81	64	28	465
(% trap success)	(9.3)	(22.6)	(8.8)	(6.5)	(3.1)	(10.1)
# rodent species	3	4	6	7	4	7
# shrews	29	27	23	29	8	116
(% bucket success)	(3.2)	(2.9)	(2.5)	(2.8)	(0.9)	(2.5)
# shrew species	2	2	2	2	2	2
**TOTAL**						
# sample-nights	1426	1426	1426	1426	1407	7111
# individuals	167	257	122	156	49	751
(% sample success)	(11.7)	(18.0)	(8.6)	(10.9)	(3.5)	(10.5)
# species	5	6	9	10	6	10

**Table 3:** Elevational distribution of shrew species along the southeastern slope of Mt. Meru in July-August 2009. Only specimens caught in traps or buckets are included.

Elevation	1950 m	2300 m	2650 m	3000 m	3600 m	Totals
Species						
*Crocidura allex*	31	31	18	36	16	132
*C*. *newmarki*	49	18	22	51	4	144
Total # individuals	80	49	40	87	20	276
Total # species	2	2	2	2	2	2
Total # sample-nights	1426	1426	1426	1426	1407	7111
Sample success (%)	5.6	3.4	2.8	6.1	1.4	3.9
Total # caught in buckets	51	22	17	58	12	160
Total # bucket-nights	506	506	506	506	495	2519
Bucket success (%)pitfall lines	10.1	4.3	3.3	11.5	2.4	6.3

**Table 4:** Elevational distribution of rodent species along the southeastern slope of Mt. Meru in July-August 2009. Only specimens caught in traps or buckets are included.

Elevation	1950 m	2300 m	2650 m	3000 m	3600 m	Totals
Species						
*Otomys tropicalis*	0	0	2	1	1	4
*Dendromus insignis*	0	0	1	7	2	10
*Grammomys dolichurus*	3	4	2	4	0	13
*Lophuromys verhageni*	0	18	9	30	2	59
*Mus triton*	0	0	0	1	0	1
*Praomys taitae*	79	185	38	4	0	306
*Rhabdomys dilectus*	0	0	24	7	24	55
*Graphiurus murinus*	5	1	6	15	0	31
Total # individuals	87	208	82	69	29	475
Total # species	3	4	7	8	4	8
Total # sample-nights	1426	1426	1426	1426	1407	7111
Sample success (%)	6.1	14.6	5.7	4.8	2.1	6.7
Total # caught in traps	86	206	81	64	28	465
Total # trap-nights	920	920	920	920	912	4592
Trap success (%)	9.3	22.4	8.8	6.9	3.1	10.1

**Fig 2:**
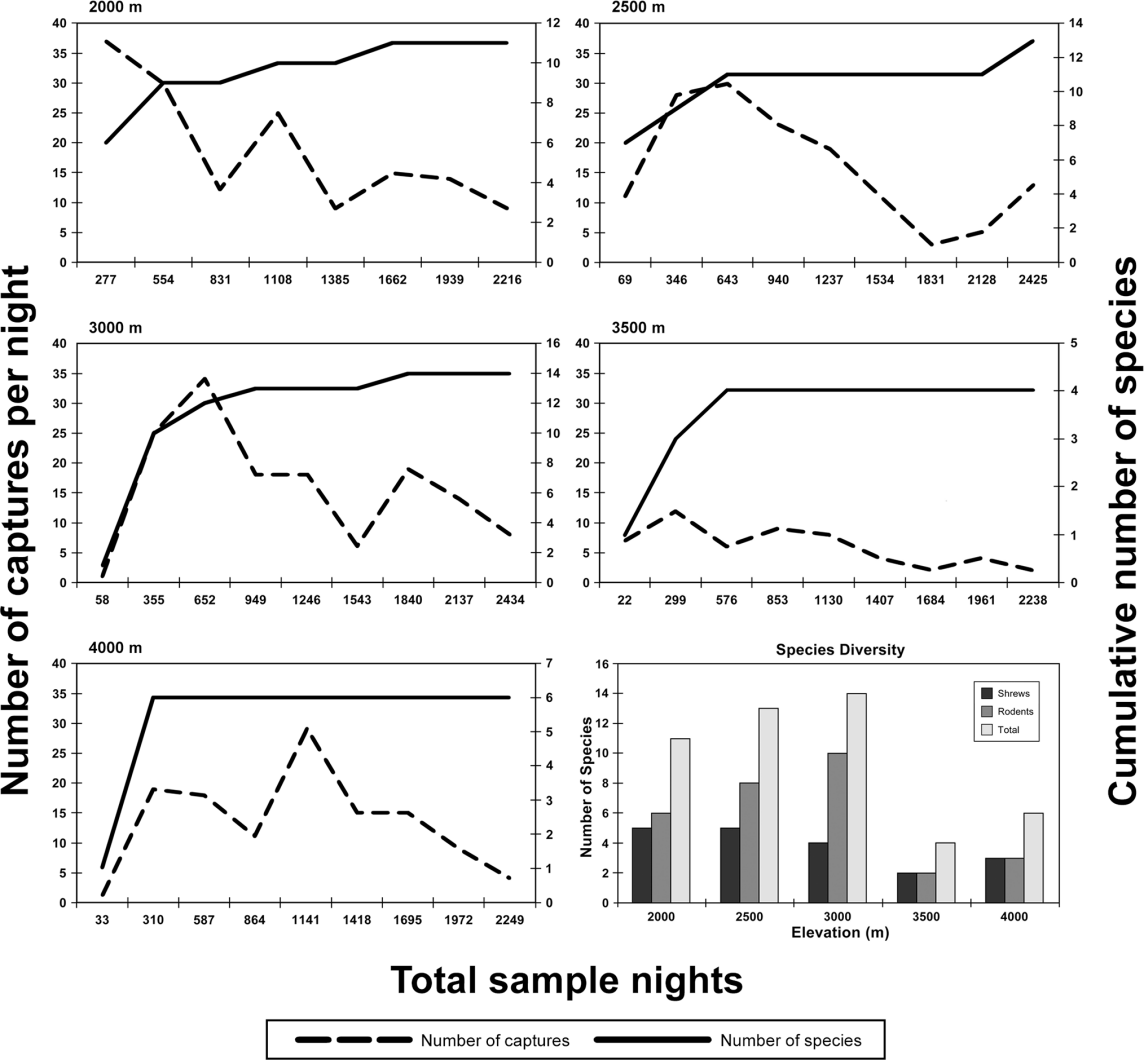
Species accumulation curves (for both pitfall and trap lines combined) for each site surveyed for small mammals on Mt. Meru. The dashed lines represent the number of captures each day; the solid lines represent the cumulative number of new species for the site observed each day. The graph at the lower right shows the number of specimens of shrew, rodent, and combined small mammals captured at each site.

At each elevational site, captures (and overall sample success) ranged from 49 [3.5%] at 3600 m to 257 [18.0%] at 2300 m (Table 2). For shrews, the lowest values were recorded at the 3600 m site (20 [1.4%]) and the highest values at the 3000 m site (87 [6.1%]; Table 3). For rodents, the lowest (29 [2.1%]) and highest (208 [14.6%]) values were observed at the 3600 and 2300 m sites, respectively (Table 4). The cumulative number of species trapped at a site reached an asymptote at the 1950, 2650, and 3600 m sites. In contrast, new species (for a given site) were captured at 2300 m, (where *Graphiurus murinus* was captured for the first time at that site on the last day of trapping) and at 3000 m, (where *Mus triton* and *Otomys tropicalis* were both captured on the last day). The species accumulation curves illustrate these results (Fig 2).

**Fig 3:**
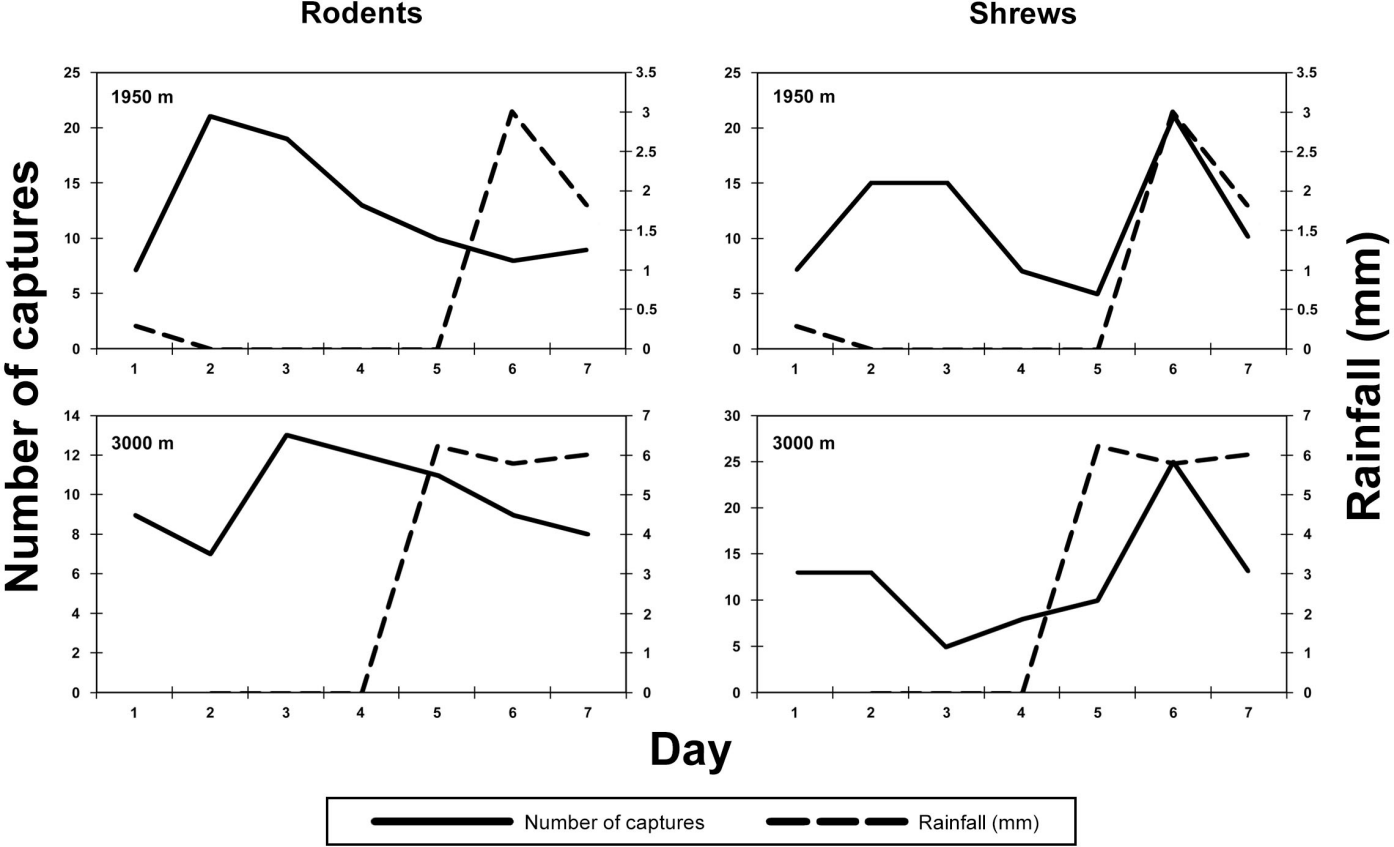
The relationship between numbers of individuals of small mammals captured each day of the sampling period, and rainfall, at each site on Mt. Meru. Rodentia are on the left and Soricomorpha are on the right.

As in past surveys of montane mammals in Tanzania [12], [14], the relationship between the amount of rainfall and shrew capture success is more pronounced than that between rainfall and rodent capture success. During the survey of Mt. Meru, only two sites (1950 and 3000 m) received rain while buckets and traps were in place. The Product-moment correlation coefficients (*r*) of amount of daily rainfall with total capture success of shrews (for buckets and traps combined) are 0.60 and 0.55 for the 1950 and 3000 m sites, respectively. For rodents these values for the same two elevational zones were both negative (-0.53 and -0.30, respectively). While none of these *r* values are significant, Fig 3 illustrates the increase in shrew captures during or shortly after measureable rainfall, a pattern not exhibited by rodentcapturess.

The correlation of four daily capture parameters (number of individuals, number of species, number of new species [i.e. previously unsampled at a given site], and cumulative number of species) with cumulative sample-nights was analyzed for both types of trapping methodology (Table 5) and mammalian order (Table 6). Because only two species of shrews were recorded during the entire survey (Tables 2 and 3), and both were caught the first day of capture and every subsequent day, correlation analysis between cumulative sampling effort and some parameters is not applicable. Other comparisons revealed differing patterns. The correlation between sampling effort and number of individuals fluctuated among elevations and the number of species captured each day was not correlated with cumulative sampling effort. For all taxa, a negative correlation existed between new species captured and cumulative sampling effort, but the correlation was significant in only five cases across the transect and not consistently based on a single parameter. Correlation analysis revealed a significant positive correlation between cumulative sample-nights and cumulative species across all sites for trap lines, bucket lines (for captures of both shrews and rodents), and both sampling methods combined. Each site exhibited the same general pattern, although not in all parameters examined. For example, there was no strong relationship between cumulative sample nights and cumulative shrew species captured in buckets at the 2650 and 3600 m sites (Table 5). Table 6 illustrates the same general pattern when the analysis is segregated by each of the two represented orders (shrews–Soricomorpha and rodents–Rodentia). Again, the low number of shrew species captured on Mt. Meru influences the analysis, and the strongest relationship is that of cumulative species of rodents with cumulative sampling effort.

**Table 5:** Product-moment correlation coefficients (*r*) of cumulative sample-nights with four parameters of trap/bucket captures associated with small mammal surveys on Mt. Meru. Results are given separately for rodents and shrews, and then all small mammals combined. Values in parentheses represent strong but not statistically significant correlations.

Daily cumulative sample-nights correlated with (across)	Number of individuals	Number of Species	New species added	Cumulative species
**Total**				
Traps (rodents only)	-0.425**	0.060	(-0.317)	0.943**
Traps (all captures)	-0.448**	0.001	-0.388*	0.920**
Buckets (shrews only)	-0.254	(-0.311)	(-0.293)	-
Buckets (all captures)	-0.226	-0.176	(-0.255)	0.910**
Traps and buckets combined (all captures)	-0.479**	-0.011	-0.350*	0.962**
**1950 m**				
Traps (rodents only)	-0.403	0.119	(-0.697)	(0.605)
Traps (all captures)	0.071	0.671	(-0.636)	0.785*
Buckets (shrews only)	-0.453	(-0.611)	(-0.605)	-
Buckets (all captures)	-0.464	-0.677	-0.784*	0.605
Traps and buckets combined (all captures)	-0.159	0.119	-0.802*	0.605
**2300 m**				
Traps (rodents only)	-0.350	0.009	-0.491	0.796*
Traps (all captures)	-0.260	0.429	(-0.691)	0.846*
Buckets (shrews only)	-0.888**	-0.774*	-0.605	-
Buckets (all captures)	-0.851	(-0.693)	(-0.677)	0.796*
Traps and buckets combined (all captures)	-0.385	-0.210	-0.576	0.796*
**2650 m**				
Traps (rodents only)	0.221	0.546	(-0.707)	0.812*
Traps (all captures)	0.336	0.627	-0.633	0.751*
Buckets (shrews only)	-0.823*	(-0.676)	-0.796*	0.605
Buckets (all captures)	-0.779*	-0.413	-0.438	0.889**
Traps and buckets combined (all captures)	0.081	(0.667)	(-0.682)	0.866**
**3000 m**				
Traps (rodents only)	0.071	0.484	-0.523	0.935**
Traps (all captures)	0.551	0.636	-0.831*	0.912**
Buckets (shrews only)	0.129	0.200	-0.605	-
Buckets (all captures)	0.090	0.293	-0.401	0.611
Traps and buckets combined (all captures)	0.453	0.255	-0.458	0.941**
**3600 m**				
Traps (rodents only)	0.285	0.588	0.144	0.975**
Traps (all captures)	0.416	(0.670)	0.000	0.971**
Buckets (shrews only)	0.309	0.224	-0.408	0.612
Buckets (all captures)	0.368	0.378	-0.196	0.849*
Traps and buckets combined (all captures)	0.501	(0.688)	-0.289	0.927**

* = p≤0.05

** = p≤0.01.

**Table 6:** Product-moment correlation coefficients (*r*) of cumulative sample-nights with four parameters of trap success shrew and rodent captures associated with small mammal surveys on Mt. Meru. Values in parentheses represent strong but not significant correlations.

Shrew and rodent captures correlated with (across)	Number of individuals	Number of species	New species added	Cumulative species
Total, shrews	(-0.290)	-0.346*	(-0.293)	-
Total, rodents	-0.421**	0.141	-0.252	0.969**
1950 m, shrews	0.141	-	-0.605	-
1950 m, rodents	-0.414	0.119	(-0.697)	(0.605)*
2300 m, shrews	-0.769*	-0.408	-0.605	-
2300 m, rodents	-0.341	-0.009	-0.491	0.796*
2650 m, shrews	-0.275	0.605	-0.796*	0.605
2650 m, rodents	-0.203	0.628	-0.611	0.900**
3000 m, shrews	0.351	-	-0.725*	-
3000 m, rodents	0.011	0.629	-0.605	0.941**
3600 m, shrews	0.644	0.408	-0. 408	0.612
3600 m, rodents	0.339	0.784*	0.000	0.980**

* = p≤0.05

** = p≤0.01.

The relationship between elevation and number of individuals or species collected, or sample success was not notable. The low and relatively constant number of shrew species was observed at all elevations, and the only prominent negative relationship (high but not significant r values) exists in the associations of the total number of individuals and total trap success with elevation (Table 7). The highest species diversity was seen at the 3000 m site and the lowest at the 1950 m site. While the lowest number of individuals collected was at the 3600 m site, the species diversity was relatively high, with 6 species, in contrast to that of the 1950 m site, with 5 species, which had the second highest sample success of any of the sites (Table 2).

**Table 7:** Product-moment correlation coefficients (*r*) between elevation and trap success associated with small mammal surveys on Mt. Meru. Values in parentheses represent strong but not significant correlations.

Elevation correlated with	(*r*)	p
Total number of individual mammals collected	(-0.75)	> 0.05
Total trap success	(-0.75)	> 0.05
Total number of species collected	0.59	> 0.05
Total number of shrews collected	-0.53	> 0.05
Shrew trap success	-0.52	> 0.05
Total number of shrew species collected	**-**	**-**
Total number of rodents collected	-0.62	> 0.05
Rodent trap success	-0.61	> 0.05
Total number of rodent species collected	0.33	> 0.05

The bucket success rates for all captured mammals was 6.7%, and 170 individuals (160 shrews and 10 rodents) were collected in 385 buckets (77 buckets installed at each of 5 sites), but most buckets captured no animals. Over the entire survey, 287 buckets caught nothing, and only98 buckets (25% of total installed) captured animals. When comparing the capture rate of individual buckets, the following figures were recorded (number of buckets–number of animals): 61 buckets—1 animal, 20–2, 10–3, 3–4, 3–5, and 1–11. A similar pattern was exhibited by traps although there was a 12.6% trap success in 750 traps with 581 captures; only 313 traps (42% of total employed) caught at least one animal. When comparing the capture rate of individual traps, the following figures were recorded (number of traps–number of animals): 166 traps– 1 animal, 74–2, 43–3, 18–4, 8–5, 2–6, and 1–7. These different values for trap success of individual buckets and traps do not follow a Poisson distribution (G-test for goodness of fit = 34.0 for buckets, 63.7 for traps; P<0.01) indicating a lack of trap independence.

## Discussion

The combination of traps and pitfall lines were effective in sampling non-volant small mammal communities at different elevations on Mt. Meru, as in past studies in Tanzania [12], [14], [22], [30], [31]. However, species accumulation curves failed to reach a plateau at every site: the 2300 and 3000 m site had one and two species, respectively, captured during the last 24-hour period of trapping. While we are relatively confident that the vast majority of shrew and small rodent species occurring at each site were documented, and are justified in making inter-site comparisons along the transect and to other elevational transects conducted in Tanzania [12], [14], we suggest additional surveys are needed on Mt. Meru to determine with certainty the complete list of small mammals in different elevation zones.

Ten mammal species (2 shrews and 8 rodents) were documented along an elevational transect from roughly 1950 m to 3600 m on the eastern slope of Mt. Meru, none of which are introduced taxa. With the exception of *Crocidura newmarki* and *Lophuromys verhageni*, which are endemic to Mt. Meru [32], [33], all species have more or less broad distributions. *Crocidura allex* is found on other east African mountains, which include Kilimanjaro, Ngorongoro, Kenya, and Aberdares [25]. Among the rodents, the species with the broadest distributions are *Grammomys dolichurus*, *Rhabdomys dilectus*, and *Graphiurus murinus*, all of which are distributed in eastern and southern Africa [28], [29]. The rodent species with the most restricted distribution, other than the local endemic *Lophuromys verhageni*, was *Praomys taitae*, which occurs from southeastern Kenya through eastern Tanzania [27].

The faunal list of Demeter and Hutterer [18] represents the heretofore most complete list of shrews and rodents known to occur on Mt. Meru, and their list contained species not observed during this survey. Some of these taxa are larger and not the subjects of the methodology employed during this study (i.e. *Paraxerus*, *Thryonomys*, and *Tachyoryctes*) and are not considered further here. The elevational range of the study of Demeter and Hutterer [18] was 1200 m to 2750 m and included towns and villages such as Arusha and Tengeru, and habitats other than forest, such as savanna. Some of the taxa they listed include taxa typically found at elevations and habitats lower than the forest on Meru, including *Mastomys natalensis* and *Pelomys fallax*. However, some taxa listed from forest localities that we did not observe are worthy of discussion here, including *Crocidura hildegardeae*, *C*. *luna*, *Aethomys kaiseri*, *Hylomyscus denniae*, *Lemniscomys striatus*, *Mus gratus*, *Rattus rattus*, and *Otomys irroratus*, The two species of Crocidura were collected at elevations of 1700 m or lower below the elevational range of the current study and we conclude that they were not present (or notably rare) at our sampled sites.

The specimens of *Aethomys kaiseri*, *Lemniscomys striatus*, and *Rattus rattus* cited by Demeter and Hutterer [18] came from habitats below 2000 m, with the exception of specimens from”House Mgondah” at 2000 m. The authors suggest *Aethomys* and *Rattus* may have occurred at this locality because of human influence, and certainly *Rattus* is a known commensal [29]. While the vast majority of our efforts were in primary habitat, we did place traps around the dwellings of Saddle Hut (not included in this analysis) to collect rodents living under the buildings. At this site, we collected *Crocidura allex*, *Lophuromys verhageni*, and *Rhabdomys dilectus* but no *Aethomys*, *Lemniscomys* or *Rattus*. We saw no evidence of these species in forested habitats of the mountain, and hypothesize that all three genera were found at this locality either having been introduced by human activities, or are supported by habitat alteration associated with the dwelling.

Demeter and Hutterer [18] list *Mus gratus* among the taxa occurring on Mt. Meru. While one locality (“Forest House”; 1700 m) associated with this species was below our study area, another (Meru East: 1550–2750 m) does overlap with our elevational transect. Given that there was no specific elevational information for the *M*. *gratus* collection sites, we cannot definitely determine if it was obtained in forest. We saw no evidence of this species, and only one specimen of *M*. *triton* was obtained during the 2009 survey.

The records of *Otomys irroratus* listed by Demeter and Hutterer [18] (now *O*. *angoniensis* [29]) are interesting to us because there are also specimens in the Field Museum collected by B. Cooper in 1938 from the crater of Mt. Meru at roughly 2900 m (FMNH 48610–48619). We recorded *O*. *tropicalis* from the same crater and at elevations ranging from 2650–3600 m, but found no evidence of *O*. *angoniensis*. Temporal (1938 vs. 2009) or seasonal (January vs. July/August) variation may explain these differences, but additional surveys of the mountain are needed to determine the current presence and distributional extent of both *Otomys* species.

The records of *Hylomyscus denniae* on Mt. Meru [18] (and Ngorongoro [34]) are of a taxon now referred to *Praomys taitae* [26], [27]. Another rodent that is found on northern Eastern Arc Mountains and the Southern Highlands [29], and has been recorded from Moshi (Dieterlen [35]), is *Beamys hindei*, and this taxon was not recorded on Mt. Meru by us or in past work. In addition to the survey of Mt. Meru, similar detailed faunal surveys have been conducted on Mt. Kilimanjaro [14] and Ngorongoro (WT Stanley, unpubl. data) and neither *Hylomyscus* or *Beamys* have been recorded on any of these Northern Highland mountains during these transects, suggesting they do not occur in these locales. Assuming this to be correct, this supports the hypothesis that the establishment of these two rodent taxa on the mountains where they are found was via a southern route. We suspect establishment via a northern route from Kenya would have resulted in populations of both of these rodents on some, if not all of the Northern Highlands.

The results of this survey differ from those on other mountains in Tanzania using identical techniques [12], [14]. The most striking difference is the low diversity of shrews on Mt. Meru, a non-Eastern Arc massif. Restricting comparisons to species lists generated by our surveys of the other Tanzanian mountains, for shrews, Mt. Meru had one genus (*Crocidura*) and two species (*C*. *allex*, *C*. *newmarki*); Mt. Kilimanjaro (non-Eastern Arc massif) had three genera (*Crocidura*, *Myosorex*, and *Sylvisorex*) and six species (*C*. *allex*, *C*. *hildegardeae*, *C*. *monax*, *C*. *olivieri*, *M*. *zinki*, and *S*. *granti*); and the Udzungwas (part of the Eastern Arc system) had three genera (*Crocidura*, *Myosorex*, and *Sylvisorex*) and nine species (*C*. *hildegardeae*, *C*. *desperata*, *C*. *elgonius*, *C*. *munissii*, *C*. *olivieri*, *C*. *telfordi*, *M*. *kihaulei*, *S*. *lixus*, and *S*. *megalura*). The Udzungwa survey included dry forest near the base of the scarp at 600 m and after removing taxa found in this zone, measures of shrew diversity drop from 9 to 7 species. Even with this reduction, the Mt. Meru shrew list stands in stark contrast to the lists of these other two massifs. The reasons for such a strikingly low shrew diversity on Mt. Meru are unknown, but given the relatively high diversity of species on Mt. Kilimanjaro and a comparable level to the Udzungwas, geological origin does not seem to explain these differences. Volcanic activity on the mountain has been recent in comparison to Mt. Kilimanjaro [36], but it seems unlikely that eruptions would have contributed to extinction of taxa once existing on Mt. Meru. Unlike Mt. Kilimanjaro and the Udzungwas, elevation was not significantly correlated with capture rates or species diversity for either shrews or rodents. In the Udzungwas, rodent diversity and abundance increased with elevation [12], and on Mt. Kilimanjaro, shrew abundance and diversity decreased with elevation [14]. Similar patterns, or any influence of elevation on abundance or diversity were not seen on Mt. Meru. As on Mt. Kilimanjaro [14], the greatest diversity of shrews and rodents on Mt. Meru was at 3000 m, and not at the top of the transect as in Udzungwa [12], and the greatest diversity was documented within forested habitats and not above tree line (Tables 2, 3 and 4).

Similarities among the Mt. Meru, Mt. Kilimanjaro, and Udzungwa transects were observed in the effect rainfall had on the capture rates of shrews, but this effect was not seen on rodents. The Mt. Meru survey adds additional support to the idea that the amount of rainfall during a survey period is strongly correlated with estimates of shrew diversity or abundance. Also, there was a lack of capture independence among traps and buckets across the entire transect at each site on Meru, as was also the case on Mt. Kilimanjaro and the Udzungwas [12], [14]. Based on these parallel results, we hypothesize that the presence of a captured animal in a bucket may attract other animals to that bucket and placement of traps influences the chances of multiple captures over time in a given trap.

Although this may change with future taxonomic studies, the only endemic mammals on Mt. Meru are *Lophuromys verhageni* [32] and *Crocidura newmarki* [33]. The *Lophuromys* was found at all sites along our transect, with the exception of the lowest (1950 m), which was unexpected, as *L*. *aquilus* is found in moist habitats on Mt. Kilimanjaro at 2000 m [14]. While the habitat at the Mt. Meru 1950 m site appeared suitable for *Lophuromys* based on our experience of trapping this genus on different Tanzanian mountains where they can be abundant [12], [14], [37], its absence is unexpected. No rodent species was recorded at all sites during the Mt. Meru survey, while both shrew species occurred along the complete transect. Much still needs to be learned about the ecology of small mammals on the high mountains of east Africa. For example, the upper limits of these animals on Mt. Meru, as well as on Mt. Kilimanjaro, remain unknown. Additional surveys are needed to determine these aspects and to further elucidate the natural history of the mammals of Meru.
