# Correction: Elevational Distribution and Ecology of Small Mammals on Tanzania's Second Highest Mountain

**DOI:** 10.1371/journal.pone.0225985

**Published:** 2019-11-26

**Authors:** William T. Stanley, Philip M. Kihaule

The corresponding author, William T. Stanley, is deceased. Philip M. Kihaule will serve as corresponding author moving forward. Dr. Kihaule’s contact information is: mammalsoftanzania@fieldmuseum.org; +255 715 144 042.

Fig 2 is incorrect. A corrected version is provided here.

**Fig 2:**
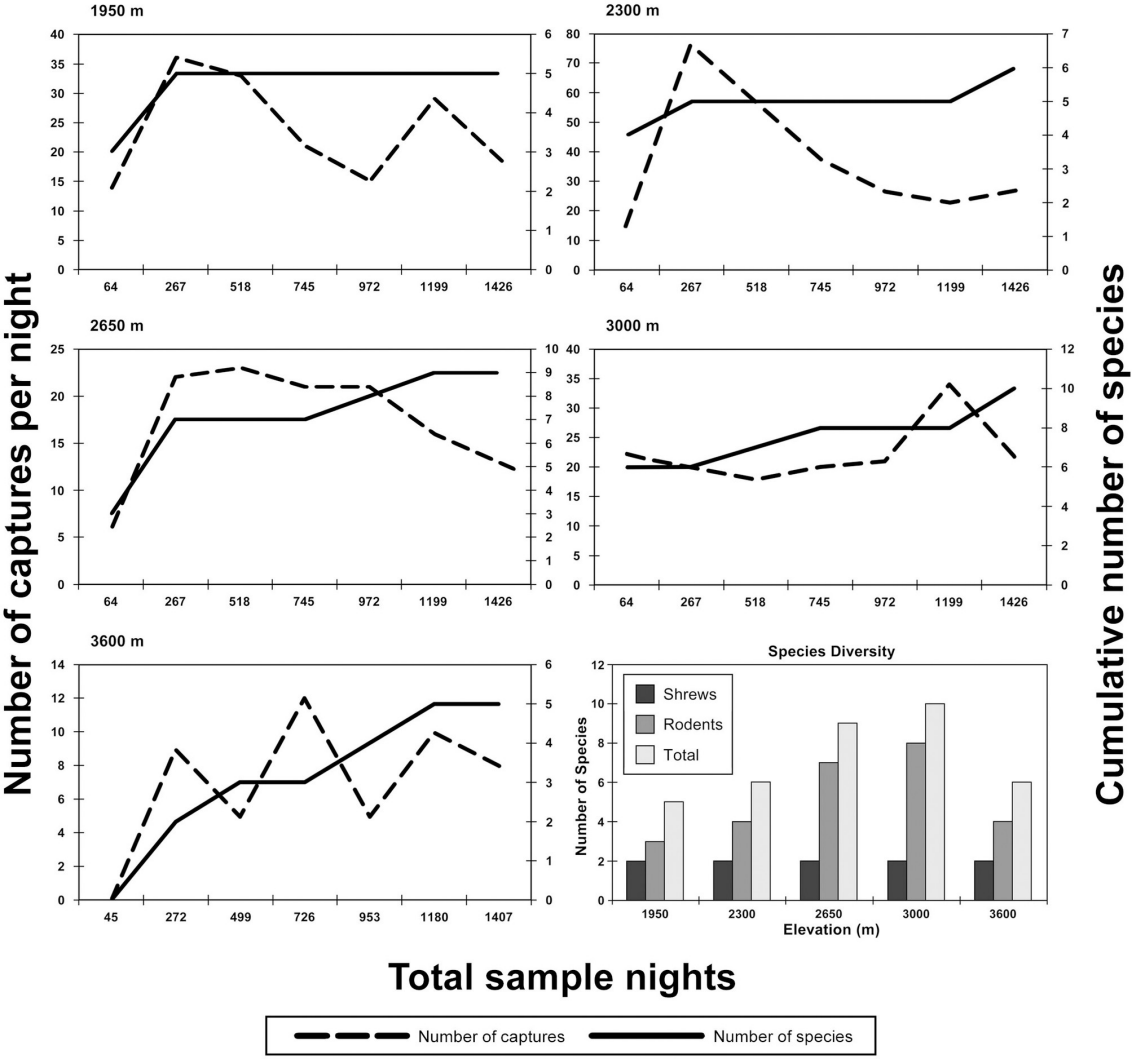
Species accumulation curves (for both pitfall and trap lines combined) for each site surveyed for small mammals on Mt. Meru. The dashed lines represent the number of captures each day; the solid lines represent the cumulative number of new species for the site observed each day. The graph at the lower right shows the number of specimens of shrew, rodent, and combined small mammals captured at each site.
